# Are we out of the woods yet? Youth-developed recommendations on recovery from the COVID-19 pandemic: A national Delphi study

**DOI:** 10.17269/s41997-025-01020-w

**Published:** 2025-04-16

**Authors:** Meaghen Quinlan-Davidson, Kristin Cleverley, Skye Barbic, Darren Courtney, Gina Dimitropoulos, Lisa D. Hawke, Nadia Nandlall, Clement Ma, Matthew Prebeg, J. L. Henderson

**Affiliations:** 1 Centre for Addiction and Mental Health, Toronto, ON Canada; 2 Dalla Lana School of Public Health, University of Toronto, Toronto, ON Canada; 3 Department of Psychiatry, University of Toronto, Toronto, ON Canada; 4 Lawrence Bloomberg Faculty of Nursing, University of Toronto, Toronto, ON Canada; 5 Faculty of Medicine, University of British Columbia, Vancouver, BC Canada; 6 Foundry BC, Vancouver, BC Canada; 7 Faculty of Social Work, University of Calgary, Calgary, AB Canada; 8 York University, Toronto, ON Canada

**Keywords:** Youth mental health and substance use, Post-pandemic recovery, Delphi study, Consensus, Santé mentale et consommation de substances chez les jeunes, Rétablissement post-pandémie, Étude Delphi, Consensus

## Abstract

**Objectives:**

To generate concrete, youth-derived recommendations to support Canada’s post-pandemic recovery from COVID-19 to support youth mental health and substance use (MHSU), economic, and educational recovery.

**Methods:**

Using a virtual, modified Delphi, participants rated recommendation items over three rounds, with the option to create their own recommendation items. A priori consensus was defined as ≥ 70% of the entire group, or subgroups of youth (e.g., age, race/ethnicity, gender and sexual identities), rating items at a 6 or 7 (on a 7-point Likert scale). Items were dropped in subsequent rounds if they did not achieve consensus. Qualitative responses were analyzed using content analysis for Round 1.

**Results:**

A total of 40 youths participated in Round 1, with good retention (97.5%) in subsequent rounds. Youths achieved consensus on eight recommendations to support post-pandemic recovery. Youths endorsed post-pandemic strategies that prioritize the implementation of effective, accessible, and low-cost MHSU services in schools, workplaces, and communities; the integration of MHSU education into school lessons; increased awareness about MHSU services in schools and workplaces; and the prioritization of health and well-being in schools and workplaces.

**Conclusion:**

Findings indicate the need for stronger partnerships between schools, community-based MHSU services, and hospitals, and job opportunities that pay a living wage.

## Résumé

**Objectifs:**

Générer des recommandations concrètes, issues des jeunes, pour soutenir la reprise post-pandémie du Canada après la COVID-19 afin de soutenir la reprise économique, éducative et de la MHSU (santé mentale et la toxicomanie) des jeunes.

**Méthodes:**

À l’aide d’une méthode Delphi virtuelle modifiée, les participants ont évalué les éléments de recommandation sur trois cycles, avec la possibilité de créer leurs propres éléments de recommandation. Un consensus a priori a été défini comme ≥ 70% de l’ensemble du groupe, ou des sous-groupes de jeunes (p. ex., âge, race/ethnicité, sexe et identités sexuelles), ayant évalué les éléments à 6 ou 7 (sur une échelle de Likert à 7 points). Les éléments ont été abandonnés lors des cycles suivants s’ils n’atteignaient pas le consensus. Les réponses qualitatives ont été analysées à l’aide d’une analyse de contenu pour le cycle 1.

**Résultats:**

Un total de 40 jeunes ont participé au cycle 1, avec une bonne rétention (97,5%) lors des cycles suivants. Les jeunes sont parvenus à un consensus sur huit recommandations pour soutenir la reprise post-pandémie. Les jeunes ont approuvé des stratégies post-pandémiques qui donnent la priorité à la mise en œuvre de services MHSU efficaces, accessibles et peu coûteux dans les écoles, les lieux de travail et les communautés; l’intégration de l’éducation MHSU dans les cours scolaires; une sensibilisation accrue aux services MHSU dans les écoles et les lieux de travail; et la priorité accordée à la santé et au bien-être dans les écoles et les lieux de travail.

**Conclusion:**

Les résultats indiquent la nécessité des partenariats plus solides entre les écoles, les services MHSU communautaires et les hôpitaux, et des opportunités d’emploi qui paient un salaire décent.

## Introduction

The Coronavirus disease (COVID-19) led to an unprecedented global challenge, creating disruptions in health, economic, and social systems. The World Health Organization declared the COVID-19 outbreak a public health emergency in January 2020 and a pandemic in March 2020 (WHO, 2024). The impact that the pandemic has had on youth (12–25 years of age) mental health and substance use (MHSU) has been particularly profound. In a meta-analysis of depression and anxiety symptoms among children and adolescents, findings showed that the overall pooled prevalence rate was 20.5% among youth, nearly double prepandemic estimates at 12.9% and 11.6% for depression and anxiety, respectively (Racine et al., 2021). A repeated cross-sectional population-based study (2015–2023) among youth 13–20 years of age in Finland showed that the prevalence of anxiety, depression, and social anxiety symptoms increased in 2023 (post-pandemic), compared to pandemic and pre-pandemic levels (Kiviruusu et al., 2024). The social and economic challenges, including reduced working hours and job losses, experienced by this population group have been associated with poor mental health and problematic substance use among youth (Parry et al., 2022).

In Canada, the federal government responded rapidly to alleviate the economic impacts of the pandemic; however, as public health restrictions eased and activities returned, less attention was paid to recovery strategies, particularly for youth (Government of Canada, 2022). Developing post-pandemic recovery strategies and policies are important to mitigate the long-term pandemic effects and future demands on the mental health system (McDaid, 2021). Particularly as MHSU conditions are associated with chronicity, the pandemic’s impact on youth mental health is projected to have lasting consequences (Patel et al., 2023). Defined as strategies that promote the return to pre-pandemic circumstances, post-pandemic recovery strategies are evidence-informed approaches that can include activities that support safety and public health, re-establish and expand essential services, and provide basic necessities, among others (Belita et al., 2022).

Prior literature on recovery responses to COVID-19 and other public health emergencies for the general population include prioritizing mental health support, community engagement, financial support, resource investment, and partnerships (Belita et al., 2022). Consultations with youth, families, and service providers on COVID-19 pandemic recovery in British Columbia revealed a need for: (i) actions to address mental health inequalities among marginalized and hard-to-reach populations; (ii) collaborative relationships between youth and mental health partners; and (iii) social and emotional learning strategies within schools and communities (Samji, 2023). While there is some evidence with respect to COVID-19 recovery strategies (Belita et al., 2022), there is a lack of information on consensus among a diverse group of youth about post-pandemic recovery recommendations relevant to them.

An essential approach to the design of post-pandemic recovery strategies is understanding youth perspectives and engaging youth throughout the recovery process (Brownlie et al., 2017). For strategies and policies to meet their needs, it is important to understand what youth value and prioritize. It also requires multidisciplinary, integrated approaches to ensure an inclusive and equitable recovery (OECD, 2022). Indeed, engaging youth in the identification of their post-pandemic recovery strategy priorities will ensure that policies, programs, and services are appropriate and relevant to this population group.

An approach that can be used to identify the priorities of youth is the Delphi method. As a consensus-building technique, the Delphi approach fully engages youth in research, prioritizing their expertise (Pike et al., 2015). Greater research is needed to better understand how youth experienced the pandemic in order to inform future post-pandemic recovery policies, services, and systems (McDaid, 2021). As such, we conducted a national Delphi study to determine youth’s COVID-19 recovery recommendations for government, policymakers, and service planners based on their pandemic experiences. The objective of this study was to generate concrete, youth-derived recommendations to support Canada’s post-pandemic recovery from COVID-19 to support youth MHSU, economic, and educational recovery.

## Methods

A virtual, modified Delphi technique was used to develop and rate recommendations, thus establishing consensus from representative perspectives on COVID-19 pandemic recovery. The Delphi technique we followed is described elsewhere (Quinlan-Davidson et al., 2024). We followed the Guidance on Conducting and Reporting Delphi Studies (CREDES) Checklist (Jünger et al., 2017) (Table 1). REDCap, an online data capture platform, was used to administer the Delphi study. The study was approved by the Centre for Addiction and Mental Health’s (CAMH) Research Ethics Board in Toronto, Canada (144/2021).

**Table 1:** Guidance on Conducting and Reporting Delphi Studies (CREDES) Checklist

Rationale for Delphi technique		**Page**
*Justification.* The choice of the Delphi technique as a method of systematically collating expert consultation and building consensus needs to be well justified. When selecting the method to answer a particular research question, it is important to keep in mind its constructivist nature		4
Planning and design		
*Planning and process.* The Delphi technique is a flexible method and can be adjusted to the respective research aims and purposes. Any modifications should be justified by a rationale and be applied systematically and rigorously		4–7
*Definition of consensus.* Unless not reasonable due to the explorative nature of the study, an a priori criterion for consensus should be defined. This includes a clear and transparent guide for action on (a) how to proceed with certain items or topics in the next survey round, (b) the required threshold to terminate the Delphi process, and (c) procedures to be followed when consensus is (not) reached after one or more iterations		7
Study conduct		
*Informational input.* All material provided to the expert panel at the outset of the project and throughout the Delphi process should be carefully reviewed and piloted in advance in order to examine the effect on experts’ judgements and to prevent bias		6
*Prevention of bias.* Researchers need to take measures to avoid directly or indirectly influencing the experts’ judgements. If one or more members of the research team have a conflict of interest, entrusting an independent researcher with the main coordination of the Delphi study is advisable		No conflicts reported
*Interpretation and processing of results.* Consensus does not necessarily imply the ‘correct’ answer or judgement; (non)consensus and stable disagreement provide informative insights and highlight differences in perspectives concerning the topic in question		7–8
*External validation.* It is recommended to have the final draft of the resulting guidance reviewed and approved by an external board or authority before publication and dissemination		7–8
Reporting		
*Purpose and rationale.* The purpose of the study should be clearly defined and demonstrate the appropriateness of the use of the Delphi technique as a method to achieve the research aim. A rationale for the choice of the Delphi technique as the most suitable method needs to be provided		4
*Expert panel.* Criteria for the selection of experts and transparent information on recruitment of the expert panel, sociodemographic details including information on expertise regarding the topic in question, (non)response and response rates over the ongoing iterations should be reported		5
*Description of the methods.* The methods employed need to be comprehensible; this includes information on preparatory steps (How was available evidence on the topic in question synthesized?), piloting of material and survey instruments, design of the survey instrument(s), the number and design of survey rounds, methods of data analysis, processing and synthesis of experts’ responses to inform the subsequent survey round, and methodological decisions taken by the research team throughout the process		4–8
*Procedure.* Flow chart to illustrate the stages of the Delphi process, including a preparatory phase, the actual ‘Delphi rounds’, interim steps of data processing and analysis, and concluding steps		6–7
*Definition and attainment of consensus.* It needs to be comprehensible to the reader how consensus was achieved throughout the process, including strategies to deal with non-consensus		7–8
*Results.* Reporting of results for each round separately is highly advisable in order to make the evolving of consensus over the rounds transparent. This includes figures showing the average group response, changes between rounds, and any modifications of the survey instrument such as deletion, addition, or modification of survey items based on previous rounds		9–12
*Discussion of limitations.* Reporting should include a critical reflection of potential limitations and their impact on the resulting guidance		14–15
*Adequacy of conclusions.* The conclusions should adequately reflect the outcomes of the Delphi study with a view to the scope and applicability of the resulting practice guidance		15
*Publication and dissemination.* The resulting guidance should be clearly identifiable from the publication, including recommendations for transfer into practice and implementation		12–15

*Source*: Jünger et al. (2017)

### Youth Expert Advisory Committee

A Youth Expert Advisory Committee was established with support from the Youth Engagement Initiative team at the Margaret and Wallace McCain Centre for Child, Youth & Family Mental Health at CAMH. We followed principles outlined by the Canadian Institutes of Health Research (CIHR) Strategy for Patient-Oriented Research (SPOR) and the McCain Model of Youth Engagement (Darnay et al., 2019). The Committee met every three to four months to actively partner on the project, and included three Youth Advisors (youth who consult on project activities) and two Youth Engagement Specialists (CAMH youth staff who support youth engagement activities and facilitate research teams and youth advisors relationships). All youth were 12–25 years of age, with lived/living MHSU-related experiences. They received honoraria for their time. The role of the Committee was to advise on study design, starting recommendations, Delphi implementation, interpretation of results, and knowledge translation products. They were ineligible to participate in the Delphi panel as research participants.

### Youth Delphi Expert Panel Members

We used two pre-existing CAMH studies and internal CAMH networks in Ontario, Canada to recruit Youth Delphi Expert Panel Members (Quinlan-Davidson et al., 2023). Eligibility criteria included youth who were 12–25 years of age, lived in Canada, and had lived/living experience of MHSU concerns at the time of the Delphi study. Following recommended Delphi panel size range (Hasson et al., 2000), we aimed to recruit *n* = 40 youths. Youths received $35 honorarium for their participation in each round of the study.

### Survey development

Qualitative responses from a pre-existing longitudinal, cohort CAMH-based study in August 2021 were used to derive the initial candidate recommendations (Hawke et al., 2021). In that study, youths responded to five open-ended questions on recovery from the COVID-19 pandemic (Table 2). For the current study, 13 starting recommendations were generated for Round 1 and presented to the Youth Delphi Expert Panel Members.

**Table 2:** Open-ended questions used to formulate Delphi Round 1 starting recommendations

	Questions
1	What do you think will be the biggest challenges that youth will face after the worst of the COVID-19 pandemic is over and we’re returning to a new normal?
2	Considering these challenges, what steps should the government and policy makers take to guide Canada’s recovery from COVID-19 in ways that support youth?
3	What steps should organizations providing mental health and substance use services take to guide Canada’s recovery from COVID-19 in ways that support youth?
4	What steps should schools and employers take to guide Canada’s recovery from COVID-19 in ways that support youth?
5	Who should be involved in making decisions about Canada’s recovery from COVID-19? How should they be involved?

### Delphi procedure

The study took place between July 2022 and April 2023, over the course of three Delphi rounds. Participants received an email at the beginning of each round, with a link to the survey. To complete each round, Panel Members ranked each recommendation item using a 7-point Likert scale (1 ‘one of the least important’ to 7 ‘one of the most important’), indicating the importance of the item. In each round, Members could provide comments and/or edits to each recommendation item in an open-ended response field. Participants also had the opportunity to create their own recommendations in Rounds 1 and 2. In Round 1, we obtained demographic characteristics of the participants (e.g., age, gender identity, race/ethnicity, province/territory).

**Fig. 1:**
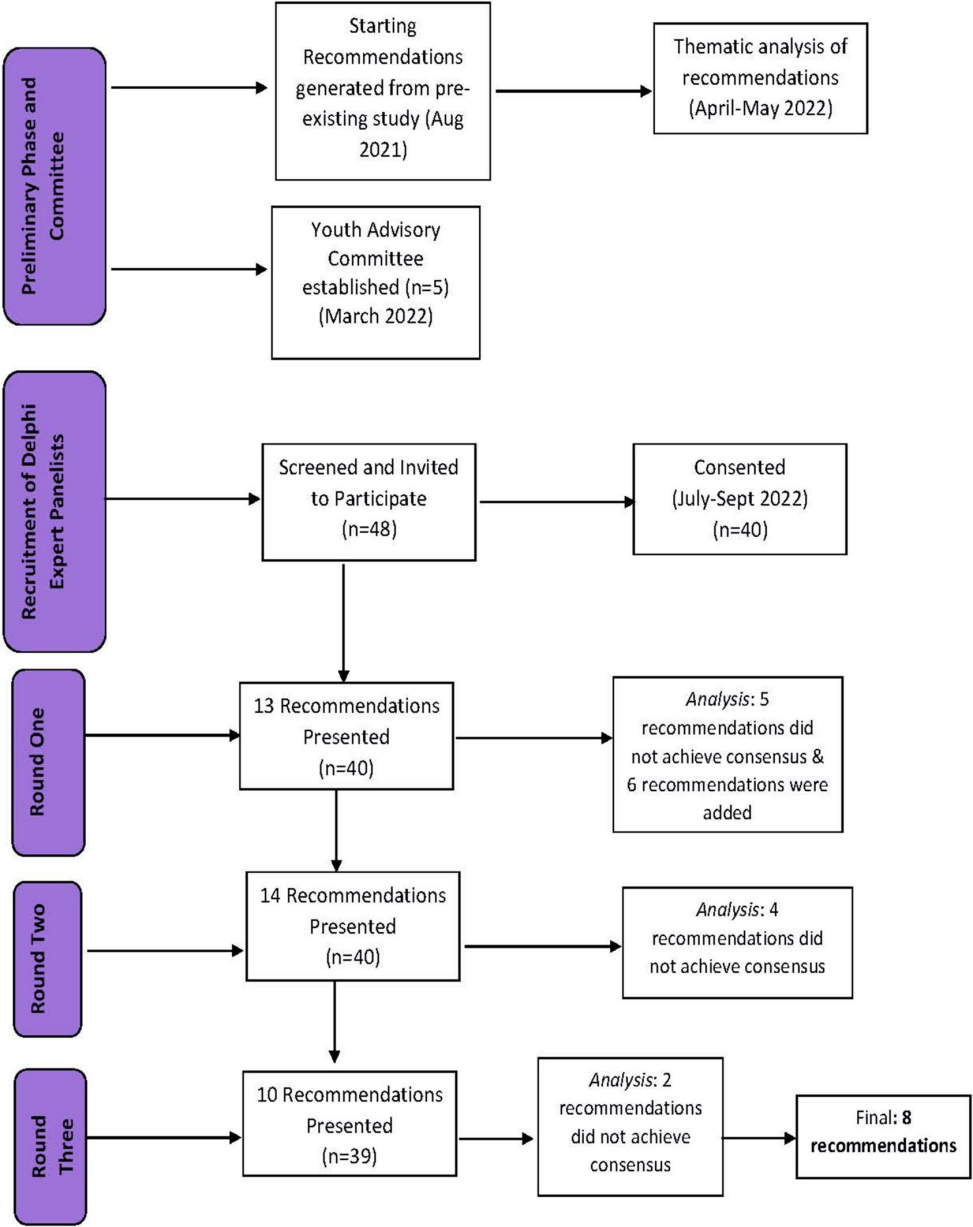
Flow diagram of Delphi rounds on COVID-19 recovery

Each round was open for three to four weeks. Reminder emails to complete the survey were sent every week. De-identified quantitative and qualitative findings from Round 1 were included in Rounds 2 and 3 (Fig. 1). The language of items that achieved consensus were reviewed by the Youth Expert Advisory Committee to ensure relevancy, appropriateness, and accessibility to youth. Revisions by the Panel Members and the Youth Expert Advisory Committee were taken forward to the next round.

### Data analysis

Descriptive statistics were used to analyze demographic data. For each round, we calculated the percentage of participants who ranked recommendations at a 6 or 7 on a 7-point Likert scale, indicating that the recommendations were highly endorsed. A priori consensus was achieved if ≥ 70% of the entire group, or subgroups of youth (identified a priori by the Youth Expert Advisory Committee as age, race/ethnicity, gender and sexual identities, and urban/rural location to determine COVID-19 priorities for these groups), rated recommendations at a 6 or 7 (on a 7-point Likert scale). Items that did not achieve consensus were dropped in subsequent rounds. Youth Panel Members were presented with their individual and group mean score of each recommendation item in the prior round and asked to re-rate the items in rounds 2 and 3. Qualitative responses in Rounds 1 and 2 were analyzed using content analysis (Burnard, 1991), with qualitative responses included in Round 1 only. We used Stata 16.1 to perform quantitative analyses.

If statements and themes were re-occurring and relevant, new recommendation items were created. The Youth Expert Advisory Committee was presented with the recommendations that achieved consensus in Round 3 to ensure consistency and relevancy of the recommendations.

## Results

In Round 1, *n* = 40 youth participated. Participation rate from Round 1 to Round 2 was 100% (*n* = 40) and from Round 2 to Round 3, 97.5% (*n* = 39). Among participants, 45%, 30%, and 25% identified as a girl/woman (cis, trans), nonbinary and gender-diverse, or boy/man (cis, trans), respectively. Forty percent of youth were from Central Canada (Ontario, Quebec) and 32.5% were from the Prairies (Table 3).

**Table 3:** Select demographic characteristics of Youth Delphi Expert Panel Members, Round 1 (*n* = 40)

Category		Mean (range)
Age (years)		20.0 (14–25)
		**n (%)**
Age category (years)	14–16	5 (12.5)
	17–21	21 (52.5)
	22–25	14 (35.0)
Gender identity	Boy/Man (cis, trans)	10 (25.0)
	Girl/Woman (cis, trans)	18 (45.0)
	Nonbinary and gender-diverse	12 (30.0)
Ethnicity	Indigenous Canadian	3 (7.5)
	Asian	13 (32.5)
	Black	1 (2.5)
	Latino	1 (2.5)
	Mixed race	1 (2.5)
	White	21 (52.5)
Sexual orientation	Straight	21 (52.5)
	2SLGBQ +	19 (47.5)
Born in Canada	Yes	31 (77.5)
	No	9 (22.5)
Region*	Prairies	13 (32.5)
	Western Canada	7 (17.5)
	Northern Territories	1 (2.5)
	Eastern Canada	3 (7.5)
	Central Canada	16 (40.0)
Area of residence	Large city or suburbs	24 (60.0)
	Small city, town, rural	16 (40.0)

^*^Region: Prairies = Manitoba, Saskatchewan, and Alberta; Western Canada = British Columbia, Northern Territories = Nunavut; Atlantic Canada = New Brunswick, Newfoundland, Nova Scotia, Prince Edward Island; Central Canada = Ontario, Quebec

### Round 1

In Round 1, Youth Delphi Expert Panel Members ranked 13 starting recommendations on level of importance (Table 4). Level of agreement was highest for Recommendations E (77.5% of all youth), F (72.5%), and M (70.0%). Rural youth (87.5%), youth 22–25 years of age (78.6%), girls/women (72.2%), and white youth (71.4%) prioritized Recommendation G. Further, rural youth (87.5%), nonbinary and gender-diverse youth (83.3%), and youth 22–25 years of age prioritized Recommendation H. Youth 14–16 years of age (80.0%) and boys/men (70.0%) prioritized Recommendation I. Meanwhile, rural youth (93.7%), 2SLGBQ + youth (73.7%), white youth and youth 17–21 years of age (71.4%, respectively) prioritized Recommendation K (Table 4).

**Table 4:** Percentage of Delphi Experts rating recommendations at 6 or 7, all rounds

Recommendations	Round 1 (*n* = 40) n (%)	Round 2 (*n* = 40) n (%)	Round 3 (*n* = 39) n (%)
A. Offer both in-person and virtual learning options to all youth in schools	Did not achieve consensus		
B. Provide students with the opportunity to return to in-person schooling at their own pace	Did not achieve consensus		
C. Provide teachers at all levels with the opportunity to participate in training that will enhance their virtual teaching skills	Did not achieve consensus		
D. Offer more socializing opportunities to youth (e.g., meet and greets, extracurricular, clubs, etc.)	Did not achieve consensus		
E. Implement free, accessible, and effective mental health and substance use services to all youth in schools, workplaces, and communities (e.g., counselling, check-ins, support groups)	31 (77.5%) of all youth**	35 (89.7%) of all youth**	34 (87.2%) of all youth**
F. Integrate mental health and substance use education into school lessons	29 (72.5%) of all youth**	33 (82.5%) of all youth**	29 (76.3%) of all youth
G. Ensure that schools, workplaces, and communities educate youth on the mental health and substance use services that they provide	15 (71.4%) white youth 13 (72.2%) girls/women 14 (87.5%) rural youth 11 (78.6%) 22–25 year old youth	29 (72.5%) of all youth	29 (74.4%) of all youth
H. Ensure that schools, workplaces, and communities educate youth on how/where to access the mental health and substance use services that they provide	10 (83.3%) nonbinary and gender-diverse youth 14 (87.5%) rural youth 10 (71.4%) 22–25 year old youth	29 (72.5%) of all youth	8 (72.7%) nonbinary and gender-diverse youth 11 (78.6%) 2SLGBQ + youth
I. Offer in-person and virtual mental health and substance use supports and allow youth to choose how they would like to receive support (e.g., group support, individualized support)	7 (70.0%) boys/men 4 (80.0%) 14–16 year old youth	9 (75.0%) nonbinary and gender-diverse youth 12 (75.0%) rural youth 10 (71.4%) 22–25 year old youth	Did not achieve consensus
J. Meaningfully include people from all age groups and communities in making decisions about pandemic recovery	Did not achieve consensus		
K. Ensure that medical and health experts (e.g., physicians, psychologists, and mental health professionals) are actively involved in making decisions about pandemic recovery	15 (71.4%) white youth 15 (93.7%) rural youth 14 (73.7%) 2SLGBQ + youth 15 (71.4%) 17–21 year old youth	12 (75.0%) rural youth	Did not achieve consensus
L. Meaningfully include youth in making decisions about pandemic recovery	Did not achieve consensus		
M. Provide youth with access to more job opportunities that pay a living wage (i.e., youth live comfortably with their needs met)	28 (70.0%) of all youth**	32 (80.0%) of all youth**	32 (82.0%) of all youth**
N. Offer more supports and accommodations in schools and workplaces (e.g., transitions back to school/work, homework help, career counselling, extra time for tests, use of assistive technology, etc.)		9 (75.0%) nonbinary and gender-diverse youth	9 (81.8%) nonbinary and gender-diverse youth 3 (75.0%) 14–16 year old youth 10 (71.4%) 22–25 year old youth
O. Provide teachers at all levels with the training and resources to effectively facilitate online learning and support students with different learning styles		Did not achieve consensus	
P. Ensure schools and workplaces prioritize the health, safety, and well-being of all their students and employees		30 (75.0%) of all youth	33 (84.6%) of all youth**
Q. Fund more socialization programs that are accessible for youth (e.g., online programs, after-school programs, extracurriculars, recreational activities, etc.)		Did not achieve consensus	
R. Provide youth employees with flexible working hours and paid mental health and sick days		30 (75.0%) of all youth	31 (79.5%) of all youth
S. Consider the impact of pandemic-related decisions on people from all age groups and communities and listen to their perspectives		Did not achieve consensus	

^**^ Denotes top recommendations in each round based on percentage highly endorsed on importance

Six recommendations did not achieve consensus and were dropped (Recommendations A–D, J, L) (Table 4). These recommendations focused on in-person and virtual schooling, socialization, and youth and communities meaningfully included in decision-making about the pandemic recovery.

Youth Delphi Expert Panel Members provided open-ended responses to create new recommendations in Round 1. Similar statements were grouped into areas, including the following: (i) school and workplace supports and accommodations; (ii) teacher training for online learning; (iii) the health, safety, and well-being of students and employees; (iv) investment in youth socialization programs; (v) flexible work hours and paid mental health and sick days; and (vi) considerations for pandemic-related decisions on all-groups and communities. Seven new recommendations were added (Recommendations N–S) based on open-ended responses during this round (Table 4).

### Round 2

In Round 2, Youth Delphi Expert Panel Members ranked 13 recommendations on importance (Table 4). Level of agreement was highest for Recommendations E (89.7% of all youth), F (82.5%), and M (80.0%). Nonbinary and gender-diverse youth and rural youth (75.0%, respectively), and youth 22–25 years of age (71.4%) prioritized Recommendation I. 75.0% of rural youth prioritized Recommendation K while 75.0% of nonbinary and gender-diverse youth prioritized Recommendation N (Table 4).

Three recommendations did not achieve consensus and were dropped (Table 4). These recommendations focused on teacher training to facilitate online learning; youth socialization programs; and the impact of pandemic-related decisions on all-groups and communities. No new recommendations were added.

### Round 3

In Round 3, Youth Delphi Expert Panel Members ranked ten recommendations on importance, while eight recommendations achieved consensus (Table 4). Level of agreement was highest for Recommendations E (87.2%), P (84.6%), and M (82.0%). Percentage of agreement among all youth was 79.5%, 76.3%, and 74.4% on Recommendations R, E, and G, respectively. 2SLGBQ + youth (78.6%) and nonbinary and gender-diverse youth (72.7%) prioritized Recommendation H. Recommendation N was endorsed by 81.8% of nonbinary and gender-diverse youth, 75.0% of 14–16 year olds, and 71.4% of 22–25 year olds.

Two recommendations did not achieve consensus and were dropped (Table 4). These recommendations focused on in-person and virtual MHSU supports and ensuring medical and health experts are actively involved in making decisions about recovery from the pandemic.

## Discussion

To our knowledge, this national study is the first to define youth-developed recommendations on post-pandemic recovery from the COVID-19 pandemic in Canada. Youth achieved consensus on eight recommendations to support COVID-19 pandemic recovery. These recommendations indicate that youth value post-pandemic strategies that prioritize the implementation of effective, accessible, and low-cost MHSU services in schools, workplaces, and communities; the integration of MHSU education into school lessons; increased awareness about MHSU services in schools and workplaces; and the prioritization of health and well-being in schools and workplaces. Youth also valued greater access to jobs that pay a living wage; flexible working hours; paid mental health and sick days; and supports and accommodations in schools and workplaces.

The recommendations generated by youth in this study align with previous recommendations on recovery responses to COVID-19 and other public health emergencies (Belita et al., 2022). Similar to previous recommendations, youth supported the prioritization of mental health support (Belita et al., 2022) within schools and communities (Samji, 2023). Youth created new recommendations, focusing on the provision of jobs that pay a living wage, paid mental health and sick days, and the need for support and accommodations in schools and workplaces. Youth are experts of their own lived experience; they know what works and what does not work (Hawke et al., 2018; Ontario Centre of Excellence for Child and Youth Mental Health, 2016), which may not be understood or represented when recommendations are generated by research experts (Triplett et al., 2022). At the same time, the Delphi method is a sound process for eliciting expert opinion (de Meyrick, 2003). Indeed, engaging youth in this process increases the relevancy, acceptability, fit, and impact of these recommendations (Hawke et al., 2018; Henderson et al., 2018).

Results of the study indicate that youth recommend implementing free, accessible, and effective MHSU services to all youth in schools, workplaces, and communities (e.g., counselling, check-ins, support groups). This finding may suggest that the current mental health support offered in schools is not effective. In fact, there is mixed evidence on the effectiveness of school mental health programs (Caldwell et al., 2019). It should be noted that this mixed evidence could be due to variability in program evaluation, intervention design, and the integration of multidisciplinary actors and organizations within these programs (e.g., schools, health and social service providers). At the same time, schools’ capacity to deliver timely and effective mental health care over the last decade has been challenging (McGartland, 2013), made even more difficult by the COVID-19 pandemic. Read et al. (2022) conducted a descriptive overview of mental health services in post-secondary institutions across the country. Most institutions provided 24-h crisis support lines, while multiple therapy sessions and clinical assessments were less common. There were also inconsistencies on school websites about mental health service information (Read et al., 2022). Greater research is needed on school mental health programs. Although the evidence is mixed, schools could be a first step in the care pathway for youth with MHSU needs. This approach would require partnerships between schools and communities. It would also require hiring community MHSU service providers to deliver school-based MHSU services, as these providers are most familiar with the needs of the community and the resources available (Vaillancourt et al., 2021).

Based on the findings of this study, there also may be a lack of available or limited awareness about community mental health supports among youth. Although 61% of youth with early MHSU needs accessed community-based mental health services across the country (Lowe et al., 2024), there is uneven service access across provinces and territories, with equity-deserving groups often not receiving the care they need (Lowe et al., 2024). To address this challenge, there have been growing calls to revise the Canada Health Act and develop a national child and youth mental health strategy (Lowe et al., 2024; Vaillancourt et al., 2021). This strategy would include integrating MHSU services within community settings (Lowe et al., 2024); increasing investment in health information systems within MHSU community-based services to collect, monitor and evaluate youth MHSU outcomes; and strengthening partnerships and collaboration between schools and community MHSU services with hospitals (Lowe et al., 2024).

At the same time, community-based MHSU services could collaborate with schools to integrate social and emotional learning programs in primary, secondary, and post-secondary training (CMHA, 2022). This approach aligns well with another recommendation important to subgroups of youth (e.g., nonbinary and gender-diverse, 2SLGBQ +) in this study, who identified a need to educate youth in schools and workplaces on how/where to access MHSU services that they provide. At the same time, by strengthening community-based provision of mental health promotion programs, this approach would provide more timely access to care and potentially help relieve pressures on the acute hospital system.

Our findings also indicate that workplace mental health is essential. In fact, mental health in the workplace has increasingly become a concern, particularly as psychologically healthy workplaces have been associated with greater job satisfaction and performance, greater motivation, and reduced turnover (APA, 2019). 2017 estimates suggest that mental health challenges cost the Canadian economy more than $50 billion annually, due to decreased productivity, absenteeism, and greater turnover, among others (Mental Health Commission of Canada, 2017). As such, having policies and practices in place to support employee mental health is important—for example, taking steps to reduce mental health stigma in the workplace, promoting workplace mental health resources, and shaping the culture of the workplace to value mental health (Wu et al., 2021).

Youth recommended providing access to more job opportunities that pay a living wage, wherein youth can live comfortably with their needs met (e.g., food, housing). Canadians have experienced financial hardships over the past few years, particularly those who identify as women, Indigenous, recent immigrants, and from lower socioeconomic status. Indeed, the Consumer Price Index increased 3.9% on an annual average basis in 2023 (following a 40-year high increase of 6.8% in 2022 and 3.4% in 2021), with price increases in every major component (e.g., transportation, gasoline, food, shelter) (Statistics Canada, 2024). Although there is debate on the conceptualization and utility of a living wage, a review (Neumark & Adams, 2001) has suggested that increases in wages for low-paid workers has been associated with some reductions in overall poverty and a small reduction in unemployment rates. To follow through on this recommendation and support youth in covering the cost of living in their community, there is a need for local, provincial/territorial, and federal leadership to advocate and implement policy changes to ensure that employers pay a living wage. For instance, youth and youth advocates could work with public, private, and community organizations to advocate to change local wage policies. In addition to increasing wages, there needs to be an increase in work benefits, revisions to income tax rates, and an increase in the supply and quality of both jobs and workers (Cooke & Lawton, 2008). At the same time, greater research is needed on a living wage and its effects on poverty and health.

Strengths of the study include authentic youth engagement; strong participation rates; and recommendations that reflect youth’s lived experiences and needs and can inform COVID-19 recovery and future recovery efforts. Limitations include the following: most participants were between 17 and 25 years of age. These recommendations may not be reflective of youth 14–16 years of age; it is important to obtain these perspectives. Participants resided mainly in Central Canada (Ontario and Quebec) and the Prairies (Manitoba, Saskatchewan, and Alberta) and may not represent youth perspectives from other provinces and territories. Further, implementation of these recommendations will vary by province and territory, as the Canadian health system is decentralized at the provincial/territorial level. Moreover, although the study design and process is framed within the context of COVID-19 and pandemic recovery, it is possible that these recommendations would have been similar pre-pandemic or several years post-pandemic and reflect youth MHSU, economic, and educational needs.

Findings from our study illustrate youths’ priorities towards post-pandemic recovery from COVID-19 and future public health recovery strategies. Future work should focus on further examination of these recommendations and translating them into policy and action. Knowledge translation (KT) is a useful approach to incorporate evidence-informed decision-making into policy and programs. Some effective KT strategies that led to a change in practice included tailored and targeted messages; access to research evidence through an online registry; and use of knowledge brokers (Dobbins et al., 2009).

## Conclusion

Results from this Delphi study can guide long-term post-pandemic and future recovery strategies and policies to support youth in Canada—particularly in terms of partnerships between schools, community-based MHSU services, and hospitals. They indicate the preferences and values among a diverse group of youth.

## Contributions to knowledge

What does this study add to existing knowledge?

- This national study is the first to define youth-developed recommendations on post-pandemic recovery from the COVID-19 pandemic in Canada.
- Youth value post-pandemic strategies that prioritize the implementation of effective, accessible, and low-cost MHSU services in schools, workplaces, and communities; the integration of MHSU education into school lessons; increased awareness about MHSU services in schools and workplaces; and the prioritization of health and well-being in schools and workplaces.

What are the key implications for public health interventions, practice, or policy?

- Schools and community-based MHSU services should work in partnership to provide mental health promotion, prevention of MHSU, and early intervention services.
- These supports and services should be linked with the provision of hospital services, or specialized care.

## Data Availability

The data used and/or analyzed during the current study are available from the corresponding author on reasonable request.
